# Neuroplastic white matter changes in patients with major depression following lysergic acid diethylamide treatment

**DOI:** 10.1016/j.xcrm.2026.102791

**Published:** 2026-05-07

**Authors:** Mihai Avram, Aurore Menegaux, Felix Müller, Hannes Zaczek, Alexandra Korda, Helena Rogg, Anna M. Becker, Laura Ley, Matthias E. Liechti, Stefan Borgwardt

**Affiliations:** 1 University of Luebeck, Department of Psychiatry and Psychotherapy, Luebeck, Germany; 2 Institute for Neuroradiology, School of Medicine and Health, TUM University Hospital, Technical University of Munich, Munich, Germany; 3 TUM-Neuroimaging Center, School of Medicine and Health, Technical University of Munich, Munich, Germany; 4 University of Basel, Department of Psychiatry, Basel, Switzerland; 5 Division of Clinical Pharmacology and Toxicology, Department of Clinical Research, University Hospital Basel, University of Basel, Basel, Switzerland

**Keywords:** lysergic acid diethylamide, LSD, psychedelics, major depressive disorder, MDD, diffusion tensor imaging, DTI, white matter, WM, fractional anisotropy, FA, neuroplasticity, antidepressant mechanisms

## Abstract

The clinical trial NCT03866252 investigates the antidepressant effects of lysergic acid diethylamide (LSD) in 61 patients with major depressive disorder (MDD) randomized to low-dose LSD (LD-LSD; 2 × 25 μg) or moderate-to-high-dose LSD (HD-LSD; 100 μg followed by 200 μg) 4 weeks apart. Although the trial reports positive clinical outcomes, underlying mechanisms remain unclear. Here, we test whether LSD alters white matter (WM) microstructure, potentially reflecting enhanced neuroplasticity. Diffusion tensor imaging data from 35 patients (17 HD-LSD) include pre- and post-intervention scans. Voxel-wise permutation tests reveal group-by-time interactions, with increased fractional anisotropy (FA) in the internal and external capsule, sagittal stratum, and fornix/stria terminalis in the HD-LSD group. In this group, post-intervention FA values correlate with improvements in depressive symptoms at 2, 6, and 12 weeks, measured using the Inventory of Depressive Symptomatology (IDS-clinician rated [C] and IDS- self report [SR]). These findings suggest that LSD-induced WM microstructural changes are associated with antidepressant effects in MDD.

## Highlights

• Moderate-to-high LSD doses increase white matter fractional anisotropy (FA) in MDD
• FA increases occur in regions linked to structural abnormalities in depression
• Post-treatment FA increases correlate with symptom relief up to 12 weeks
• Low-dose LSD does not produce measurable changes in white matter microstructure

## Graphical abstract

**Figure:**
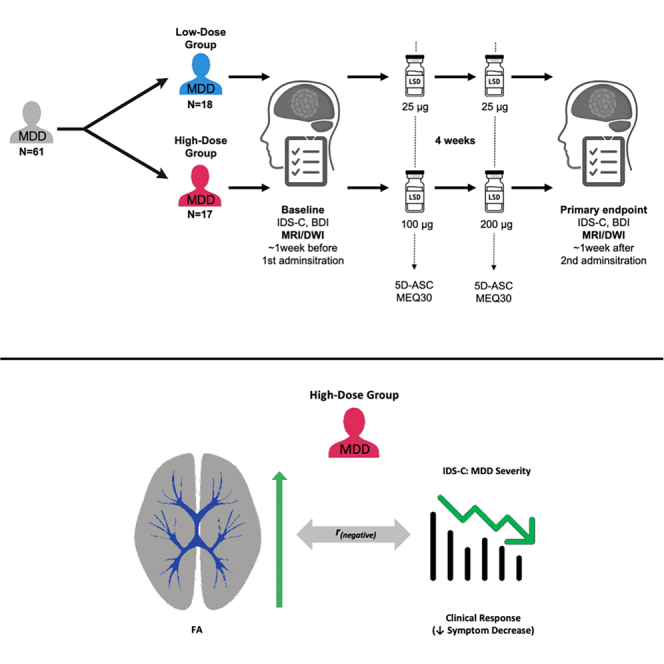

## Teaser

Avram et al. demonstrate that moderate-to-high doses of LSD, but not low doses, increase white matter fractional anisotropy in patients with major depressive disorder. These structural changes in key fiber tracts correlate with sustained clinical improvement, suggesting that LSD-induced neuroplasticity facilitates its antidepressant effects.

## Introduction

Major depressive disorder (MDD) stands as a prevalent and profoundly debilitating psychiatric condition. Its far-reaching impact on both individuals and society at large is undeniable, imposing a substantial burden in terms of healthcare expenditures, diminished productivity, and personal suffering.^1^ While various treatment options for MDD exist, approximately one-third of patients do not experience remission or substantial improvement when treated with conventional antidepressant therapies.^2^

Emerging research suggests that the symptoms of MDD may, in part, stem from maladaptive neuroplasticity. The concept of neuroplasticity, encompassing both maladaptive and corrective facets, has gained substantial traction in recent years, emerging as a promising avenue for alleviating the burden of MDD.^3^^,^^4^ Indeed, numerous treatment modalities have been investigated for their potential to induce neuroplastic changes capable of ameliorating depressive symptoms. For instance, antidepressant medication has been shown to boost adult hippocampal neurogenesis in patients with MDD.^5^^,^^6^ Moreover, antidepressants appear to normalize brain-derived neurotrophic factor (BDNF) levels in patients with MDD, a factor pivotal in nurturing the growth and survival of neurons and in fostering neuroplasticity.^7^ Electroconvulsive therapy (ECT) has also demonstrated its ability to induce neuroplasticity through processes such as synaptogenesis, neurogenesis, and dendritogenesis.^8^ An intriguing entrant in this realm is ketamine, a dissociative anesthetic agent. Ketamine has attracted significant attention due to its rapid antidepressant effects, believed to be mediated, at least in part, by molecular and cellular neuroplastic alterations.^9^^,^^10^ Despite these encouraging findings, the limited efficacy of available treatment options for MDD has spurred the search for alternatives. Psychedelics, including substances such as psilocybin, lysergic acid diethylamide (LSD), and N,N-dimethyltryptamine (DMT), have recently emerged as promising candidates. Several modern clinical trials have underscored their efficacy in alleviating depression in patients with MDD, including treatment-resistant depression (TRD).^11^^,^^12^^,^^13^^,^^14^^,^^15^^,^^16^ Among these, LSD is of particular interest due to its distinct pharmacological profile and historical precedence. Historical perspectives and early modern research have indicated that LSD possesses antidepressant properties.^17^^,^^18^ For instance, Gasser and colleagues noted that reductions in depressive symptoms mirrored the significant improvements observed in anxiety in patients with life-threatening disease. While modern trials have shown that other psychedelics, such as psilocybin and ayahuasca, are also efficient in reducing depressive symptomatology (for an overview of phase 2 clinical trials, see^19^), it remains unclear whether any one psychedelic holds a therapeutic advantage over others. This uncertainty stems from a lack of head-to-head comparisons; however, such differences are plausible given their distinct neuropharmacology, as the role of receptors beyond 5-HT2A in driving therapeutic outcomes is not yet clarified.^20^ Given its clinical efficacy, LSD has recently moved into phase 3 clinical trials (e.g., NCT06941844), bringing it closer to potential regulatory approval. Despite this clinical progress, the underlying mechanisms remain to be fully elucidated. Notably, psychedelic-induced antidepressant effects are conjectured to arise through neuroplastic changes (e.g., increases in synapses and neurite outgrowth), as demonstrated by *in vitro* and *in vivo* animal studies.^10^^,^^21^^,^^22^^,^^23^ However, evidence of *in vivo* neuroplastic changes in humans remains scarce.

Neuroimaging techniques, such as structural and functional magnetic resonance imaging (MRI), have been employed to probe treatment-induced changes in brain macrostructure (e.g., hippocampal volume) and microstructure (e.g., white matter [WM]), changes thought to arise from cellular neuroplasticity.^24^ In this context, we note that WM is not merely a passive conduit for neural signals but an active modulator of gray matter (GM) function. At the cellular level, WM influences synaptic plasticity by regulating conduction velocity and the timing of action potential propagation.^25^ In addition, intact WM is essential for metabolic and trophic support of axons, including the provision of metabolic substrates (e.g., lactate), forming a functional axo-myelinic unit; disruption of this unit can impair cellular homeostasis and lead to downstream synaptic alterations in connected GM regions.^26^^,^^27^ At the systems level, WM microstructure provides the anatomical scaffold that constrains large-scale functional connectivity, as demonstrated for the default mode network (DMN).^28^ Against this background, techniques that are sensitive to WM microstructural properties are well suited to capture biologically meaningful changes with potential downstream relevance for GM function and large-scale network organization. Diffusion tensor imaging (DTI), a technique that quantifies the diffusion of water molecules in the brain, provides a valuable means to assess changes in WM microstructure, as the diffusion characteristics are intrinsically linked to the size and orientation of WM fibers.^29^^,^^30^^,^^31^ Key DTI metrics include fractional anisotropy (FA) and mean diffusivity (MD), which respectively assess the degree of anisotropy and the average mobility of water molecules. A robust body of evidence corroborates the assertion that MDD is associated with discernible WM alterations, predominantly characterized by widespread reductions in FA and increases in MD.^32^^,^^33^^,^^34^ Several therapeutic interventions currently available for MDD have demonstrated their capacity to “normalize” the WM alterations, more or less consistently (for a review, see^35^). For example, some studies indicate that antidepressants reduce MD^36^ and increase FA in certain WM bundles among responders.^37^ Nevertheless, other studies have failed to find significant relationships between antidepressant treatment and MD or FA.^38^ Repetitive transcranial magnetic stimulation (rTMS) also appears to increase FA post-treatment in patients with MDD.^39^^,^^40^ Similarly, ECT elevates FA in individuals with MDD,^41^ although findings concerning MD are mixed.^41^^,^^42^ Notably, ketamine has also been shown to increase FA within WM regions;^43^ however, other studies have failed to observe significant effects.^44^ These findings highlight the potential interplay between clinical improvement and concurrent WM microstructural changes.

The primary objective of this study was to examine microstructural WM changes occurring after LSD treatment in individuals with MDD. Our data were derived from a recent clinical trial that compared the antidepressant effects of two low doses (LD) and two moderate-to-high doses of LSD in patients with MDD.^16^ Briefly, this study provided evidence that the two moderate-to-high doses of LSD were significantly more effective in alleviating MDD symptoms. Our hypothesis posited that these higher doses of LSD would result in increased FA and decreased MD in individuals with MDD. We assessed putative relationships between FA and MD changes in symptoms using correlation analysis.

## Results

### Participant demographics and clinical characteristics

DTI data were available for 35 patients with MDD (17 in the HD-LSD group) following quality checks. For a detailed patient description, see Table 1. Briefly, patient groups did not differ significantly in age or sex. However, independent-sample *t* tests revealed that patients in the HD-LSD group had more severe depression than those in the LD-LSD group at baseline, as evaluated by the Inventory of Depressive Symptomatology Clinician-Rated and Self-Report (IDS-C/SR) and Beck Depression Inventory (BDI). These findings align with the results reported by Müller and colleagues^16^ in the larger sample. Consistent with this, we found that at the primary endpoint (i.e., 2 weeks after the 2^nd^ intervention/week 9), patients in the HD-LSD group had a greater reduction in baseline-adjusted depressive symptoms than those in the LD-LSD group, as evaluated using both IDS and BDI. These results indicate that two moderate-to-high doses of LSD are more efficient in reducing depressive symptoms than two LD.

**Table 1:** Participant demographics and clinical characteristics

	LD-LSD	HD-LSD	Group difference	Effect size (Cohen’s d)
**N**	18	17	–	–
Age	38.1 ± 11.7	41.8 ± 12.3	*p* = 0.36	–
Sex (assigned at birth)	6/12 F/M	7/10 F/M	*p* = 0.63	–
IDS-C Week 2	26.2 ± 8.28	35.3 ± 11.1	*p* = 0.009∗	–
IDS-C Week 9 - Week 2	−1.76 ± 16.2 (*N* = 17)	−17.4 ± 10.3	*p* = 0.002∗	1.15
IDS-C Week 13 - Week 2	−2.71 ± 16.6 (*N* = 17)	−17.9 ± 13.5 (*N* = 16)	*p* = 0.007∗	1
IDS-C Week 19 - Week 2	−6.12 ± 14.2 (*N* = 17)	−16.7 ± 14.6 (*N* = 16)	*p* = 0.04∗	0.73
IDS-SR Week 2	26.94 ± 9.35	34.7 ± 11.2	*p* = 0.03∗	–
IDS-SR Week 9 - Week 2	−2.35 ± 16.83 (*N* = 17)	−17.2 ± 10.6	*p* = 0.004∗	1.05
IDS-C Week 13 - Week 2	−3.24 ± 17.3 (*N* = 17)	−17.4 ± 13.5 (*N* = 16)	*p* = 0.01∗	0.91
IDS-C Week 19 - Week 2	−7.24 ± 15.01 (*N* = 17)	−16.3 ± 14.9 (*N* = 16)	*p* = 0.09	0.60
BDI Week 2	18.9 ± 9.30	26.41 ± 10.31	*p* = 0.03∗	–
BDI Week 9 - Week 2	−2.35 ± 14.4 (*N* = 17)	−17.8 ± 9.25	*p* < 0.001∗	1.28
BDI Week 13 - Week 2	−2.24 ± 17.2 (*N* = 17)	−16.8 ± 10.60 (*N* = 16)	*p* = 0.007∗	1.01
BDI Week 19 - Week 2	−5.53 ± 13.0 (*N* = 17)	−16.1 ± 10.55 (*N* = 16)	*p* = 0.01∗	0.88

Continuous variables are presented as mean ± standard deviation (SD). Group differences for demographic variables (age, sex) were evaluated using independent samples *t* tests and chi-square tests, respectively. For clinical outcomes, group differences represent the comparison between the moderate-to-high-dose LSD (HD-LSD) and low-dose LSD (LD-LSD) groups. IDS-C (Inventory of Depressive Symptomatology – Clinician-rated) and IDS-SR (Self-Report) were defined as co-primary clinical outcomes. BDI (Beck Depression Inventory) was utilized as a secondary supportive measure. Change scores represent the difference from baseline (week 2). Effect sizes are reported as Cohen’s d. *p* values in this table represent raw (uncorrected) significance levels for the clinical comparisons.

### Group-by-time interactions in DTI-derived measures

**Figure 1:**
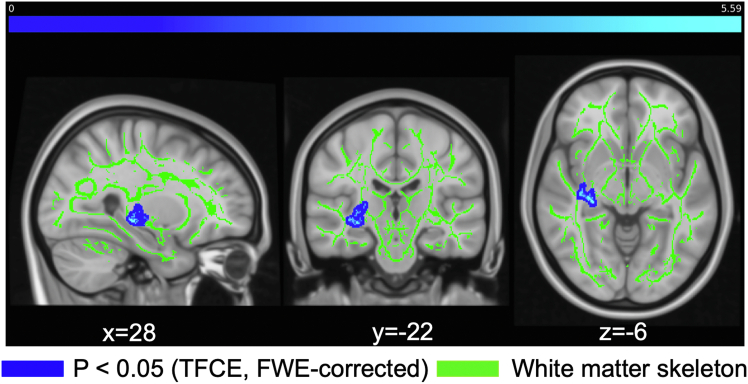
Post-intervention changes in FA following LSD treatment: Interaction effect (HD-LSD > LD-LSD) Depicted are the results of a permutation-based independent-sample *t* test on the post-intervention minus pre-intervention FA “difference images,” reflecting a group-by-time interaction between the moderate-to-high-dose (*n* = 17) and low-dose (*n* = 18) LSD groups. The identified FA clusters at *p* < 0.05 (corrected for multiple comparisons) were filled (i.e., with tbss_fill) for visualization purposes. Increases in WM microstructure were observed in the HD-LSD group in several regions (blue voxels), including the retrolenticular part and posterior limb of the internal capsule, the external capsule, the sagittal stratum, and the fornix/stria terminalis. Color intensity reflects *t*-statistic values (peak *t* = 5.59). Statistical significance was determined via 5000 permutations. The mean FA skeleton (green) is overlaid on FSLeyes’s standard MNI152_T1_0.5mm template. Coordinates are shown in MNI space. Abbreviations are as follows: FA, fractional anisotropy; TFCE, threshold-free cluster enhancement; FEW, family-wise error; MNI, Montreal Neurological Institute.

To identify group-by-time interactions, we conducted voxel-wise difference maps for each participant by subtracting the pre-intervention DTI maps from the post-intervention maps. Group differences in these difference maps were evaluated using permutation-based two-sample *t* tests. No significant group-by-time interactions were observed for MD, axial diffusivity (AD), and radial diffusivity (RD). However, we found a significant group-by-time interaction for the FA map (P_FWE_ < 0.05, threshold-free cluster enhancement (TFCE) family-wise error-corrected), with a peak *t* statistic of 5.59. Significant interactions were found in the internal and external capsule, sagittal stratum, and fornix/stria terminalis, with larger FA values in the HD-LSD group (Figure 1; Table S1). To quantify the effect size of these changes, we extracted the mean FA values from the significant cluster identified in the voxel-wise analysis and computed an independent *t* test on the change scores from pre-to post-intervention (ΔFA) between the two groups. We observed a large effect size for the FA change in the HD-LSD versus LD-LSD group (t_33_ = −4.37, *p* < 0.001, Cohen’s d = 1.48).

**Figure 2:**
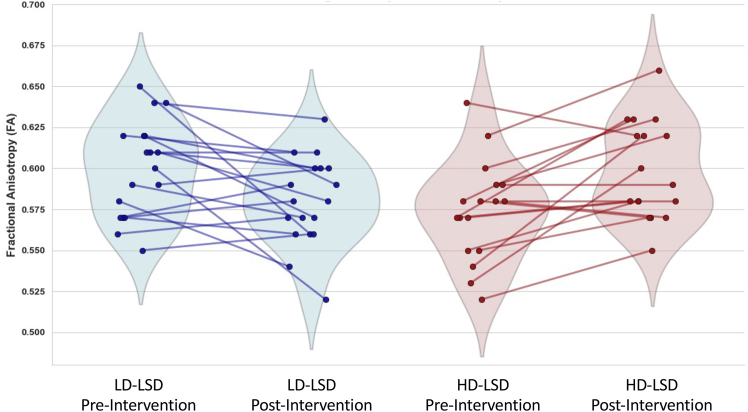
Individual trajectories of FA change by dose group following LSD treatment Displayed are the pre- and post-intervention FA values for each individual participant in the low-dose LSD (LD-LSD) group (left) and the moderate-to-high dose LSD (HD-LSD) group (right). Each line connects an individual’s pre-intervention FA value to their post-intervention FA value, extracted from the combined white matter regions showing significant group differences in the independent-samples *t* test (as shown in Figure 1). Upward-sloping lines indicate an increase in FA, while downward-sloping lines indicate a decrease. This visualization highlights the predominantly increasing FA trajectories in the HD-LSD group compared to the more variable and generally less pronounced changes in the LD-LSD group. Data processing and calculation of individual response rates were performed using an AI-assisted Python script (see Data S1).

While group-level analyses revealed overall increases in FA in the HD-LSD group, it is also insightful to examine the individual trajectories of FA changes. As depicted in Figure 2, individual patients within the HD-LSD group generally showed an increase in FA from pre-to post-intervention, particularly in the regions identified as having significant group differences. By contrast, the LD-LSD group exhibited more varied and often negligible changes in individual FA values. This finding supports the distinct impact of moderate-to-high doses of LSD on WM microstructure.

### Control analyses

To test whether the increased FA values observed in the HD-LSD group were indeed related to treatment (i.e., higher dose), we ran several control analyses*.* First, as individual values of pre-intervention FA appeared to differ visually between the two groups in Figure 2, we statistically evaluated the baseline FA values localized in the regions exhibiting group differences in the voxel-wise Group × Time interaction. An independent-samples *t* test on these specific clusters revealed that the LD-LSD group started with significantly higher baseline FA in these regions compared to the HD-LSD group (t_33_ = 2.55, *p* = 0.015). However, subsequent analysis demonstrated this to be a localized effect, as a voxel-wise, whole-brain independent-samples *t* test on the pre-intervention FA maps showed that the two groups did not differ significantly in whole-brain FA prior to the intervention. Second, we tested whether the increased post-intervention FA was influenced by age and sex. We therefore repeated the independent-sample *t* test on the post-intervention minus pre-intervention “difference maps” but included age and sex as covariates of no interest in the model (i.e., in the FMRIB Software Library [FSL] design matrix). Controlling for age and sex did not change the results, with the HD-LSD groups still showing increased FA in several WM bundles (see Figure S2).

Finally, we ran several analyses to study the effects of variation in time (i.e., days to scan) between the second intervention and the second scan: (i) an independent-samples *t* test demonstrated that the two groups did not differ in the duration between the second intervention and the second scan (t_33_ = 0.01; *p* = 0.99); (ii) a regression analysis using the number of days to scan as a predictor for post-intervention FA indicated a trend toward significance across groups (F_1,33_ = 3.76, *p* = 0.06), suggesting a possible relationship between changes in FA and time since treatment.

### Clinical scores before and after treatment

Both HD- and LD-LSD groups showed a numerical reduction in depressive symptomatology at the primary endpoint (i.e., at week 2; Table 1), although the reduction was only significant in the HD-LSD group in this reduced dataset. As the HD-LSD group had higher symptom severity at baseline, we adjusted the clinical scores at the primary endpoint and follow-ups to the baseline scores. The HD-LSD group had a significantly larger symptom reduction, as measured by the IDS-C, than the LD-LSD group at the primary endpoint and at both follow-up sessions. Similar results were also observed for IDS-SR and BDI, indicating that the larger symptom reduction was independent of the clinical scale.

### Correlations between DTI-derived measures and clinical scores

**Figure 3:**
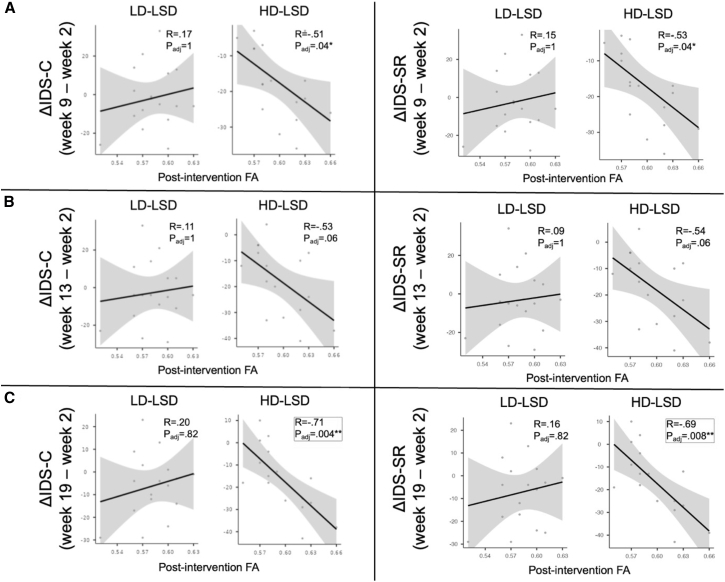
Association between post-intervention FA and depressive symptom reduction Depicted are correlations between changes from baseline (Δ) in Inventory of Depressive Symptomatology Clinician-Rated (IDS-C; left) and Self-Report (IDS-SR; right) and post-intervention fractional anisotropy (FA) values in the areas identified by the independent-samples *t* test for both the low-dose (LD) and moderate-to-high-dose LSD (HD-LSD) groups. (A) Correlations at the primary endpoint (week 9), (B) correlations at the first follow-up (week 13), and (C) correlations at the final follow-up (week 19). Blue frames around R and *p* values reflect results that remain significant after controlling for age and sex in partial correlation analyses. P_adj_ values reflect Bonferroni-Holm adjusted *p* values for the two primary endpoints. We did not correct for the various time points, as these longitudinal follow-up analyses were exploratory in nature.

Finally, we investigated whether the observed FA changes in the post-intervention maps were associated with the observed changes in depressive symptomatology. To this end, we correlated FA values from the areas showing significant group differences in the independent-samples *t* test for all participants and correlated these values to the changes in depressive symptoms from baseline. Post-intervention FA values were significantly correlated with improvements in depressive symptoms at the primary endpoint (ΔIDS-C: R = −0.51, P_adj_ = 0.04; Figure 3A, left) and at the final follow-up (ΔIDS-C: R = −0.71, P_adj_ = 0.004; Figure 3C, left) and showed a trend toward significance at the first follow-up (ΔIDS-C: R = −0.53, P_adj_ = 0.06; Figure 3B, left) in the HD-LSD group only. To test for possible influences of age and sex, we followed these analyses with partial correlations, controlling for these factors. For ΔIDS-C, the relationship with post-intervention FA was no longer significant at the primary endpoint and first follow-up but remained significant at the final follow-up (R_p_ = −0.68; P_adj_ = 0.014). For IDS-SR, results were similar and are depicted in Figures 3A–3C, right.

To test for the potential influence of the clinical scale, we also examined correlations between post-intervention FA values and ΔBDI. The results were similar to those for IDS-C/SR (see supplemental Figures S3A–S3C).

Interestingly, compared to the HD-LSD group, the LD-LSD group showed a distinct relationship between post-intervention FA and symptom change, which was not significant (Figure 3).

To further ensure that the observed associations were not driven by baseline differences in depression severity, we conducted partial correlations controlling for baseline clinical scores. While the correlations at the primary endpoint were attenuated, the relationship between post-intervention FA and symptom improvement remained robust and significant at the final follow-up for all clinical scales (see Table S5). These findings suggest that higher post-intervention FA is a significant marker for the long-term maintenance of clinical improvement, independent of the initial severity of depressive symptoms.

To control for additional possible influences, we conducted a multivariate analysis of covariance (MANCOVA) with several variables, including group (HD-LSD and LD-LSD), pre-intervention FA, age, and sex as independent variables and ΔIDS-C and post-intervention FA as dependent variables. Table S2 summarizes the results of the MANCOVA, displaying the relationships between each independent variable and the two dependent variables. Briefly, LSD dosage (i.e., group) significantly influences both the reduction in depressive symptoms (i.e., ΔIDS-C; F_1,29_ = 12.087, *p* = 0.002) and mean FA post-treatment (F_1,29_ = 5.250, *p* = 0.029), indicating a potential correlation between dosage and improvements in both mental health and brain microstructural changes. Importantly, pre-intervention FA did not have a significant relationship with the reduction in depressive symptoms (F_1,29_ = 0.723, *p* = 0.402), suggesting that this effect is related to the treatment rather than pre-intervention factors. Furthermore, while pre-intervention FA was a significant covariate in the model for post-intervention FA (F_1, 29_ = 11.677, *p* = 0.002), it did not diminish the significant main effect of dosage, suggesting that the microstructural differences observed post-treatment were driven by the intervention rather than pre-existing baseline variations.

### Correlations between DTI-derived measures and subjective effects

Subjective experience, assessed via oceanic boundlessness (OB) from the 5-Dimensional Altered States of Consciousness Questionnaire (5D-ASC) and total 30-item Mystical Experience Questionnaire (MEQ30) score, was not correlated with post-intervention FA, either across groups or within the subgroups. See Table S3 for details.

As both post-intervention FA and subjective effects were independently correlated with clinical outcome (Table S3), we further examined which factor better predicted symptom improvement (ΔIDS-C at week 2) using multiple linear regression analysis. In the full sample, OB emerged as a significant predictor (*p* = 0.002), whereas FA was not (*p* = 0.14). The model accounted for a significant proportion of variance (F_2, 38_ = 6.85, *p* = 0.003). By contrast, in the HD-LSD group, post-intervention FA significantly predicted clinical outcome (*p* = 0.030), while OB showed a similar but non-significant trend (*p* = 0.105). No significant predictors were identified in the LD-LSD group (*p* = 0.52).

These findings highlight a differential pattern of associations, with acute subjective experience better predicting outcome in the full sample, whereas post-intervention FA was more predictive in the high-dose group.

## Discussion

The present study compared the effects of two low (25 μg) and two moderate-to-high doses of LSD (100/200 μg) on DTI-derived measures in patients with MDD. Comparative analysis revealed a distinct effect, whereby the administration of two moderate-to-high LSD doses significantly increased FA in various WM bundles, including the internal and external capsule, sagittal stratum, and fornix/stria terminalis. Furthermore, a significant correlation emerged between increased FA values within these WM bundles after the interventions and a reduction in depressive symptoms. This correlation was specific to the moderate-to-high dose LSD (HD-LSD) group, persisting from the primary endpoint through follow-up assessments. While these findings are limited by the small sample size (*N* = 35) and the potential influence of baseline severity differences, they indicate that neuroplastic-like changes within WM bundles are observed after the administration of two moderate-to-high doses of LSD in patients with MDD. The implications of these results are 2-fold: first, they provide evidence of structural modifications ensuing from psychedelic compounds—putatively driven by cellular neuroplasticity—within the human brain *in vivo*; second, they propose a plausible mechanism by which the antidepressant effects of LSD might be engendered, hinting at the neurological underpinnings of its therapeutic impact.

### Moderate-to-high doses of LSD increase FA in patients with MDD

Our primary finding revealed a significant group-by-time interaction for FA but not for MD, AD, or RD, indicating a significant increase in FA over time, specifically within the HD-LSD group compared to the LD-LSD group. This finding was independent of age, sex, and pre-intervention FA values, as confirmed by comprehensive control analyses. The increased FA was observed in several subcortical WM bundles, which are pivotally involved in interconnecting distinct brain regions crucial for emotional regulation, memory processing, and executive functions. For instance, the internal capsule connects the cortex with the basal ganglia, the thalamus, and the brainstem,^45^ and is an important component of thalamocortical circuitry.^46^ The external capsule provides cholinergic pathways to the cortex, which are relevant for executive functions and emotion regulation.^47^^,^^48^ The sagittal stratum links the cortex with the thalamus and the brainstem and plays a role in visual processing and executive function.^49^ Finally, the fornix/stria terminalis links the hippocampus and amygdala to other subcortical structures such as the basal forebrain, striatum, and thalamus^50^ and is highly relevant for emotion regulation.^51^ Based on the functional roles of these subcortical WM bundles, particularly in emotion regulation, it is not surprising that alterations in these regions are often reported in patients with MDD.^34^^,^^48^^,^^52^^,^^53^ Therefore, the observed increase in FA following LSD in patients with MDD suggests a possible “normalization” or restoration of WM structural alterations, which may be crucial for therapeutic outcomes. Notably, we observed divergent trajectories for FA between the two groups, with a significant increase in the HD-LSD group and a slightly downward trend in the LD-LSD group. This finding points toward a dose-dependent threshold for triggering measurable plasticity in WM microstructure. Our control analyses support the interpretation that, while the downward trend in the LD-LSD group likely reflects regression to the mean, given their higher localized baseline in these specific regions, the consistent increase in the HD-LSD group reflects a dose-dependent effect. These findings argue against technical artifacts such as repeated scanning, which would be expected to affect both groups similarly.

The concept of treatment-induced WM changes, particularly in FA, is not unique to psychedelics and has been described following other therapeutic options for MDD known to influence neuroplasticity. For example, some studies have reported increased FA following antidepressant treatment with paroxetine in MDD responders^37^ and after ECT in regions such as the cingulum and forceps minor.^41^ Similarly, rTMS has been associated with FA increases in cerebellar and prefrontal fiber bundles.^39^^,^^40^ However, it is important to note that findings across these conventional treatments are not always consistent, with several studies failing to replicate such FA changes.^38^^,^^42^^,^^54^^,^^55^ These collective studies, despite their inconsistencies, demonstrate that brain microstructure, particularly FA, can be modulated by diverse interventions for MDD, underscoring its potential as a biomarker for treatment-response-related neuroplasticity.

In this context, our study provides evidence of longitudinal WM microstructural changes following LSD administration. While the broader landscape of human neuroimaging studies on structural changes following psychedelics remains limited, with a recent review highlighting a scarcity of robust evidence,^56^ our findings provide a direct longitudinal characterization of these effects. Previous human reports include both cortical thinning and thickening in long-term ayahuasca users.^57^ More directly relevant, a recent preprint reported a decrease in AD in prefrontal-subcortical tracts after psilocybin in healthy volunteers.^58^ Notably, Lyons and colleagues interpreted this result as potential pruning of connections or neurogenesis leading to under-myelinated axons. By contrast, our findings of increased FA in patients with MDD, a population often characterized by WM abnormalities,^34^ may better align with the concept of normalization or restoration of WM integrity, which may be crucial for therapeutic outcomes.

This interpretation is supported by a growing body of animal studies that reveal direct cellular and microstructural neuroplastic effects of psychedelics. For instance, single doses of N,N-DMT or 2,5-dimethoxy-4-iodoamphetamine (DOI) increase the density of dendritic spines on cortical neurons in adult rats, dependent on serotonin 2A receptor (5-HT2A receptor) and mTOR activation.^10^ Similarly, increased synaptic density—as measured by SV2A—was observed in the hippocampus and prefrontal cortex of pigs one week after a single dose of psilocybin.^59^ Crucially, LSD administered to chronically stressed mice reversed stress-induced reductions in medial prefrontal cortex (PFC) dendritic spines, accompanied by a reversal of anxiety- and depressive-like behaviors.^60^

### Increases in FA following LSD administration correlate with symptom relief

Our second main finding was a significant and specific correlation between post-intervention FA values extracted from the regions showing increased FA in the HD-LSD group and symptom relief. Specifically, we found that post-intervention FA values correlated with ΔIDS-C/SR at 9 weeks (i.e., 2 weeks after the second treatment) in the HD-LSD group. Remarkably, the correlation between post-intervention FA and symptom relief remained significant at both follow-up sessions, indicating long-term changes. Furthermore, these associations were not specific to the clinical scale, as changes in the BDI were also associated with FA increases. Interestingly, the association between FA and clinical improvement became increasingly robust over time, remaining significant at the final follow-up even after controlling for baseline severity.

These correlations were specific to the HD-LSD group. By contrast, in the LD-LSD group, the relationship between the two metrics appeared positive; however, it was not significant. This discrepancy in the observed relationship between the HD-LSD and LD-LSD groups highlights the complex nature of the dose-response relationship. While it might seem intuitive that the same substance, administered at different doses, would yield similar effects that differ only in magnitude, our findings suggest otherwise. The dose-response relationship for psychedelics appears not to be linear,^61^^,^^62^ and a threshold effect may underlie the differences observed in our study. Indeed, previous research has suggested that above a certain threshold dosage (i.e., related to the level of 5-HT2A receptor stimulation), distinct brain properties and subjective effects may appear.^63^ The MANCOVA analysis supports this interpretation, revealing significant associations between LSD dosage (group) and both symptom relief (ΔIDS-C) and post-intervention FA. Specifically, LSD dosage significantly influenced the reduction in depressive symptoms and changes in mean FA post-treatment, indicating a potential correlation between dosage and improvements in both mental health and brain microstructural changes. Moreover, our analysis revealed that pre-intervention FA did not have a significant relationship with the reduction in depressive symptoms, suggesting that this effect was related to the treatment (i.e., dosage) rather than pre-intervention factors.

Finally, exploratory analyses demonstrated that both FA changes and acute subjective effects may contribute to clinical improvement following LSD administration, but their relative predictive strength appears to vary by dose. In the full sample, OB emerged as a stronger predictor of symptom reduction than post-intervention FA, pointing to the relevance of acute subjective experience in mediating early treatment effects. By contrast, in the HD-LSD group, FA changes were more closely associated with clinical outcome, suggesting that structural neuroplasticity may play a more prominent role at higher psychedelic doses. These dose-dependent differences raise the possibility that distinct mechanisms—experiential vs. neurobiological—may differentially contribute to treatment response depending on LSD dose. However, these conclusions must be interpreted cautiously given the relatively small sample size, particularly within subgroups.

### Mechanistic insights

Our study’s observed WM microstructural changes following LSD treatment are well situated within the emerging framework of psychedelic-induced neuroplasticity, suggesting a potential mechanism for the observed improvements in depressive symptoms. The observed increase in WM microstructure (i.e., captured by FA) may be one of the mechanisms that provides anatomical support for the increases in brain connectivity reported by other studies. Neuroplastic changes in WM have been previously described in humans following activity-dependent (e.g., learning) or context-dependent (e.g., environmental or social) factors, and several cellular mechanisms have been proposed to support them, including myelin formation, changes in myelin thickness, modulation of internode length, and alterations in the nodes of Ranvier.^25^

Beyond activity- and context-dependent factors, antidepressant medications such as selective serotonin reuptake inhibitors (SSRIs) and ketamine have also been associated with increased myelination, plasticity, and myelin repair,^64^^,^^65^^,^^66^ supporting the link between synaptic changes and WM neuroplasticity. A key mediator in this process is BDNF, which has been shown to induce myelination in WM pathways in both animal and *in vitro* studies.^67^ Importantly, BDNF’s supporting role in myelination occurs not only during development but also during repair; evidence indicates that BDNF and other agonists of tropomyosin receptor kinase B (TrkB)—the high-affinity signaling receptor for BDNF (e.g., tricyclic dimeric peptide-6)—increase the proportion of myelinated axons, promote thicker myelin sheaths, and increase the number of post-mitotic oligodendrocytes.^68^

Recent mechanistic evidence has proposed that LSD and other psychedelics may drive neuroplastic changes by directly binding to TrkB with high affinity, thereby facilitating BDNF-mediated structural plasticity.^22^ However, this direct binding mechanism remains a subject of active debate; recent high-throughput kinase screenings and cellular reporter assays failed to find evidence of a direct physical interaction between LSD and TrkB or other human kinases.^69^

Despite these differing molecular findings, a convergent theme emerges regarding WM remodeling. For instance, ketamine can restore altered myelination by promoting the differentiation of oligodendrocyte precursor cells (OPCs) into mature oligodendrocytes.^70^ Whether LSD drives similar WM changes through a direct effect on TrkB or through downstream signaling pathways—similar to ketamine—remains an essential area for future investigation. However, ketamine’s facilitation of myelination appears to be mediated by α-amino-3-hydroxy-5-methyl-4-isoxazolepropionic acid receptor (AMPAR) signaling, which may partly overlap with the effects of psychedelics.^56^ These findings emphasize the importance of further investigating the therapeutic potential of WM changes observed following LSD administration and their comparability to conventional antidepressant treatments and ketamine.

On a more cautionary note, interpreting FA is challenging due to the measure’s dependence on multiple factors, including axon density, myelination, and cellular organization.^71^ Therefore, we are not able to argue which of the above-mentioned cellular processes are influenced by LSD. Nevertheless, our results suggest that time may play a role in modulating FA increases, as indicated by a trending correlation between the number of days to the post-intervention scan and FA increases across both groups. This finding is consistent with the duration required for changes to occur (e.g., in myelin thickness).^25^ However, the relationship between FA and myelin thickness is complex, and FA cannot directly indicate myelin changes due to its sensitivity to other microstructural factors.^72^ Hence, FA should be interpreted as a non-specific marker in myelin dynamics. For instance, while relationships have been observed between FA and myelin-related factors (e.g., myelin basic protein), these correlations vary widely across distinct WM tracts.^73^ This variation is likely driven by other factors that contribute to anisotropy, such as axonal integrity and cellular organization. For example, in a cuprizone-induced demyelination model, while FA decreased during demyelination and increased during remyelination, these shifts were also affected by changes in axonal density and organization beyond myelin dynamics.^74^ Consequently, identifying the exact cellular mechanisms leading to the observed FA increases following LSD is currently not possible using DTI alone.^25^ Future studies combining distinct neuroimaging modalities may provide additional details regarding the underlying cellular mechanisms.

### Clinical and therapeutic implications

Our findings, demonstrating specific increased FA in WM microstructural changes in patients with MDD following moderate-to-high dose LSD administration, carry significant clinical and therapeutic implications, particularly within the evolving landscape of psychiatric treatment, where current therapies often face limitations in efficacy and sustainability. The neuroplastic effects observed in our study significantly correlated with sustained improvements in depressive symptoms, suggesting a biologically plausible mechanism by which psychedelics such as LSD may exert their therapeutic effects. This notion is further supported by research indicating that pre-existing WM deficits observed in unmedicated patients with MDD may be reversible with antidepressant use,^34^ notably in regions found to be affected in our study—i.e., the sagittal stratum, external capsule, and fornix. This suggests that LSD may facilitate normalization or restoration of compromised WM integrity in the depressive brain. Furthermore, the sustained antidepressant response observed alongside these structural changes further positions LSD as a promising compound for MDD, potentially offering long-lasting benefits from a limited number of administrations.

Beyond the restoration of WM integrity, our findings provide a structural perspective that complements prior functional imaging reports. Specifically, the microstructural changes observed here align with research by Daws and colleagues,^75^ who demonstrated that decreased brain modularity (reflecting a shift toward increased global network integration) correlates with symptom relief in patients with MDD following psilocybin administration. While the acute psychedelic state is characterized by a temporary departure from structural constraints—manifested as a significant increase in between-network functional connectivity and a reduction in network integrity^76^^,^^77^^,^^78^—the persistence of these integrated network configurations in the weeks following treatment likely requires enduring physiological changes.

We speculate that the microstructural WM remodeling observed in the present study may provide a physical substrate that supports the maintenance of these “healthier” functional states. This hypothesis is consistent with the principle of structure-function correspondence, where intrinsic functional connectivity patterns are constrained and shaped by the underlying structural architecture.^28^ In this framework, the strengthening of WM tracts may reinforce integrated signaling pathways, potentially preventing a reversion to the pathologically modular network states characteristic of depression. Such findings highlight the potential for LSD to drive a coupled structural and functional recovery, warranting further investigation into the temporal dynamics of these changes.

In summary, our study provides insights into the effects of LSD treatment on WM microstructure in patients with MDD. We observed increases in FA following moderate-to-high-dose LSD administration, which were associated with symptom relief. These findings suggest a potential role for WM alterations as treatment targets in MDD, aligning with emerging evidence from neurobiological and clinical studies. Finally, the results indicate a potential structural neuroplastic mechanism associated with the antidepressant effects of LSD.

### Limitations of the study

Several limitations warrant consideration in our study. First, a significant limitation is the relatively small sample size (*N* = 35), with only 17 participants in the HD-LSD group. This limited power restricts the generalizability of our findings and increases the potential for type 2 errors; specifically, it may have hindered our ability to detect more subtle microstructural effects, particularly in the LD-LSD group. Future studies with larger cohorts could offer increased sensitivity to detect potential subtle effects. Nevertheless, the sample investigated here is similar in size to those of other recent studies investigating the effects of psychedelics in patients with mental disorders. Furthermore, we did not have a specific *a priori* hypothesis that LSD treatment would uniquely affect a single DTI metric. Consequently, we examined both FA and MD as primary outcomes, while AD and RD were treated as exploratory, without explicitly controlling for multiple testing across these metrics. This approach may increase the risk of type 1 error, and our findings should therefore be interpreted with caution. Second, it is important to acknowledge the baseline differences in illness severity between patients in the HD-LSD and LD-LSD groups. This imbalance introduces the possibility that regression to the mean contributed to the greater reduction in clinical scores observed in the HD-LSD group. While the correlation between structural WM changes and clinical improvement suggests a biological link, the lack of a placebo arm and baseline differences in symptom severity mean that these results should be interpreted as associations rather than definitive causal effects. Although we attempted to statistically control for these differences by comparing changes from baseline (also see Table S4), more severe MDD may manifest with more extensive anatomical and physiological alterations.^79^ However, the comparison of FA maps from the pre-intervention phase suggested that the groups did not differ in this regard. Third, the heterogeneity concerning the duration between the second intervention and the post-intervention scan is noteworthy. The groups did not differ in this regard, but we observed a trend toward a positive correlation between duration (i.e., number of days) and post-intervention FA (*p* = 0.06; see Figure S4). Given that neuroplastic changes, such as myelin remodeling, require time to consolidate and become detectable with DTI, this trend may reflect the temporal dynamics of WM changes following the intervention. A critical question remains whether the observed increases in FA reflect a direct pharmacological consequence of LSD treatment or a secondary effect of symptom alleviation. Since state-dependent factors related to depression recovery—such as improved sleep quality^80^ or reduced neuroinflammation^81^—can influence FA, it is possible that LSD-induced clinical improvement indirectly facilitated these structural changes. Future studies that adhere to standardized longitudinal time frames (e.g., at 1-week, 2-week, and 3-week intervals) could provide valuable insights into the temporal dynamics of FA changes and underlying cellular processes. Finally, beyond the FA limitations outlined in the previous sections, examining regions of the brain where multiple fiber bundles intersect could potentially affect the accuracy of FA.^82^ Furthermore, the inherent limitations of Tract-Based Spatial Statistics (TBSS) also need to be acknowledged.^83^ For instance, TBSS imposes certain constraints on FA values, particularly limiting them to a predefined threshold (e.g., 0.2) for skeletonization steps. This may lead to challenges in identifying FA in regions with lower FA values, such as tract perimeters. Future studies may benefit from complementing TBSS with alternative imaging techniques to overcome these limitations and provide a more comprehensive assessment of WM microstructure.

## Resource availability

### Lead contact

Further information and requests for resources should be directed to and will be fulfilled by the lead contact, Dr. Mihai Avram (mihai.avram@uksh.de).

### Materials availability

This study did not generate new unique reagents or materials.

### Data and code availability

• The datasets used in this study are derived from the NCT03866252 trial and are not publicly available due to patient privacy considerations. To request access, the requester must clearly describe the research objectives. Data access will be considered for non-commercial, research-oriented purposes only. To ensure participant privacy, access to personally identifiable information or sensitive clinical details will not be provided. All data access requests must adhere to participant consent agreements and comply with all applicable institutional and national regulations.
• This study used standard FSL processing pipelines and a custom Python script for statistical interaction analysis and visualization. The custom code used to generate the results reported in this study is available in the Supplemental information.
• Any additional information required to reanalyze the data reported in this paper is available from the lead contact upon request.

## Acknowledgments

This work was supported by the 10.13039/501100001711Swiss National Science Foundation (grant no. 32003B_185111 to M.E.L. and grant no. 320030_170249 to M.E.L. and S.B.). A.M. was supported by the Deutsche Forschungsgemeinschaft (ME 5894/2-1).

## Author contributions

M.A. conceptualized the imaging study. F.M. and M.E.L. designed the clinical study. F.M., L.L., and S.B. coordinated the clinical study. Treatments were provided, and data were collected by F.M. and H.Z. The clinical data were directly accessed, verified, and analyzed by F.M., M.E.L., and A.M.B. A.M. aided with DTI analyses. A.K., H.R., and S.B. contributed critical feedback on the manuscript and figure design. M.A. wrote the first draft. All authors critically revised and approved the final manuscript and take full responsibility for its content.

## Declaration of interests

M.E.L. acts as a consultant to Mind Medicine Inc.

### Declaration of generative AI and AI-assisted technologies

During the preparation of this work, the authors used ChatGPT-4 (OpenAI, https://chat.openai.com/) to assist with improving language clarity and the development of data processing scripts for result visualization. All AI-generated content was reviewed, modified as needed, and approved by the authors, who take full responsibility for the final version of the manuscript.

## STAR★Methods

### Key resources table

**Table:** 

REAGENT or RESOURCE	SOURCE	IDENTIFIER
**Chemicals, peptides, and recombinant proteins**		

Lysergic acid diethylamide (LSD)	Lipomed	N/A

**Software and algorithms**		

FSL (FMRIB Software Library)	FMRIB, University of Oxford	https://fsl.fmrib.ox.ac.uk
Tract-Based Spatial Statistics (TBSS)	FMRIB, University of Oxford	https://fsl.fmrib.ox.ac.uk
Jamovi	The jamovi project	https://www.jamovi.org
Python script	This paper	Supplementary Data S1

### Experimental model and study participant details

#### Clinical trial

Data were derived from the randomized, double-blind, low-dose-controlled, parallel-group, longitudinal phase II clinical trial NCT03866252, conducted in Basel, Switzerland.^16^ The trial was approved by the Ethics Committee for Northwest/Central Switzerland and by the Federal Office of Public Health. After receiving a complete description of the study, all participants gave their written informed consent.

#### Participants

Patients with MDD (*N* = 61) were recruited for the clinical trial NCT03866252. Inclusion criteria were a DSM-5 diagnosis of MDD, a score between 24 and 46 on the Inventory of Depressive Symptomatology Clinician-Rated (IDS-C), and between 26 and 48 on the IDS Self-Report (IDS-SR), thereby excluding extreme cases of MDD. For detailed participant description see.^16^

Patients were randomly assigned to one of two groups, receiving either two low-doses of LSD (25 μg; LD-LSD) or two moderate-to-high doses of LSD (100 μg in the 1^st^ intervention and 200 μg in the 2^nd^ intervention; HD-LSD), 4 weeks apart. Participants investigated in the current neuroimaging study included 35 individuals from the larger cohort, with the LD-LSD group (*n* = 18) having a mean age of 38.1 ± 11.7 years and consisting of 6 females and 12 males. The HD-LSD group (*n* = 17) had a mean age of 41.8 ± 12.3 years, with 7 females and 10 males. The primary outcome measure was changes from baseline in IDS-C and IDS-SR at 2 weeks after the 2^nd^ intervention (i.e., at week 9). For details on procedure see.^16^

Acute subjective effects were assessed after each LSD session using the 5 Dimensions of Altered States of Consciousness questionnaire (5D-ASC)^84^ and the Mystical Experience Questionnaire (MEQ30).^85^ For subsequent analyses, we focused on “oceanic boundlessness” (OB) from the 5D-ASC and the total MEQ30 score from the second LSD session, given their previously established relationship with clinical outcomes.^86^

In this study, ‘sex’ refers to sex (assigned at birth) as recorded in the clinical trial screening documentation. Information on gender identity was not collected.

### Method details

#### MRI acquisition

Magnetic resonance imaging (MRI) data, including diffusion-weighted imaging (DWI), were acquired ∼1 week before the 1^st^ LSD administration and at ∼1 week after intervention (range: 1–33 days; mean: 9.43 ± 8.66 days). 38 MDD patients had viable DWI-data, of which 35 were included in this study (see below). Neuroimaging was optional; therefore, the number of participants who underwent MRI was lower than the number of participants participating in the trial.

#### Imaging parameters

DWI-data were acquired on a 3T Siemens Magnetom Prisma scanner (Siemens Healthcare) with a 20-channel phased-array head coil. DWI data were acquired using a single-shot spin-echo echo-planar imaging sequence, resulting in one non-diffusion-weighted image (b = 0 s/mm2) and 64 diffusion-weighted images (b = 800 s/mm2, 64 non-collinear gradient directions) covering the whole brain with the following parameters: echo time (TE) = 71 ms, repetition time (TR) = 7500 ms, flip angle = 90°, a field of view (FoV) = 256 × 256 mm^2^, matrix = 128 × 128, 62 transverse slices, and voxel size = 2.0 × 2.0 × 2.0 mm^3^. Additionally, a pair of non-diffusion-weighted images with reverse phase encoding were acquired to correct for distortions.

#### Preprocessing and data quality check

DWI data were preprocessed in FSL with the FMRIB Diffusion Toolbox (www.fmrib.ox.ac.uk/fsl). The first step of preprocessing was to correct for susceptibility-induced distortions, which was performed with FSL’s TOPUP command. The following preprocessing steps included eddy current and head motion correction with outlier replacement, by registering the DWI images to the b0 image corrected for distortions, and removal of the skull and non-brain tissue, performed with FSL’s Brain Extraction Tool (BET). Subsequently, a voxel-wise tensor model was applied (FSL’s DTIFIT) from which the FA and MD maps were derived.^87^

Raw and preprocessed DWI data were visually inspected for excessive head motion and visible artifacts. Additional quality checks were performed on the preprocessed data, including visual inspection, and FSL’s QUAD and SQUAD methods (see Figure S1). Data corrupted by artifacts (e.g., motion-induced, ghosting – insufficient fat suppression, extreme distortion) were also identified using the fitting residuals – the sum-of-squared-error maps generated with FSL’s DTIFIT. Experienced radiologists at the University of Basel evaluated potential WM lesions or abnormalities; they also examined fluid-attenuated inversion recovery (FLAIR) images, which were acquired as part of the standard clinical routine.

Three participants were excluded based on corrupted data, two in the LD-LSD group – one due to extreme motion and one due to strong distortion, which could not be corrected - and one in the HD-LSD group – due to extreme motion.

### Quantification and statistical analysis

#### Tract-based spatial statistics

Voxel-wise statistical analyses for FA and MD were carried out using Tract-Based Spatial Statistics (TBSS).^88^ First, the FA images were non-linearly registered and aligned to the FMRIB58 FA template (1 × 1 × 1 mm^3^) and subsequently averaged to generate a mean FA image. This image was used to create a white matter skeleton across all subjects, which was then thresholded to FA > 0.2 to keep the main white matter tracts only. To obtain individual FA maps, each subject’s FA image was projected onto the skeleton. Finally, the tbss_non_FA tool was used to register and warp the MD, AD, and RD images following the same procedure as for FA.

#### Statistical analyses

The final sample used for the DTI analysis included 35 subjects (Table 1). Group differences in sex were computed with the Chi^2^ test and independent-sample *t* tests were used to calculate group differences in age and clinical variables at baseline (i.e., IDS-C/SR, BDI).

Statistical analyses for the DTI measures were performed with FSL’s General Linear Model (Glm; https://fsl.fmrib.ox.ac.uk/fsl/fslwiki/GLM). To assess group-by-time interactions, we computed within-subject difference maps (post-intervention minus pre-intervention) for each DTI metric. Group differences in these difference maps were then assessed using permutation-based two-sample *t* tests using FSL’s randomise with 5000 permutations.

To correct for multiple comparisons, we used threshold-free cluster enhancement (TFCE) and family-wise error correction, both with a statistical threshold of *p* < 0.05.

The John Hopkins University (JHU) ICBM-DTI-81 WM labels atlas^89^ was used to identify regions that were statistically different between the two groups. These regions were then masked and binarized to extract (i) values from areas reflecting group differences for correlation analyses and (ii) the number of voxels contained in each label.

Finally, we used Pearson’s correlation analysis to test for associations between the extracted values from areas reflecting group differences and changes from baseline in ΔIDS-C/SR and ΔBDI at 2, 6, and 12 weeks after intervention.

#### Control analyses

We performed several control analyses to ensure that our outcomes were not influenced by various parameters. To ensure that group-by-time interactions or group differences in the ‘difference images’ were not driven by differences in the pre-intervention maps, we calculated group differences in pre-intervention maps only. As an additional control analysis, we added sex and age as covariates of no interest in the independent-sample *t* tests. To evaluate the possible effects of age and sex on the associations between DTI-derived metrics and changes in symptom severity, we performed partial correlations controlling for these factors. Finally, since several parameters (i.e., dose, pre-intervention FA, age, and sex) could alter the relationship between post-intervention FA and symptom relief, we conducted a multivariate analysis of covariance (MANCOVA) to evaluate possible influences.

#### Additional resources

This clinical trial is registered at ClinicalTrials.gov.

Registry Number: NCT03866252.

Link: https://clinicaltrials.gov/study/NCT03866252.

## Supplemental information

Document S1. Figures S1–S4 and Tables S1 and S2 and Data S1

Document S2. Article plus supplemental information
