# Supplementary material for: Neuroplastic white matter changes in patients with major depression following lysergic acid diethylamide treatment
Source: Cell Rep Med. 2026 May 7;7(6):102791. doi: 10.1016/j.xcrm.2026.102791 (PMC13293959; doi:10.1016/j.xcrm.2026.102791)
Supplement: Document S1. Figures S1–S4 and Tables S1 and S2 and Data S1 [file mmc1.pdf]

**Cell Reports Medicine, Volume *7***

## Supplemental information

# Neuroplastic white matter changes in patients with major depression following lysergic acid diethylamide treatment

**Mihai Avram, Aurore Menegaux, Felix Müller, Hannes Zaczek, Alexandra Korda, Helena Rogg, Anna M. Becker, Laura Ley, Matthias E. Liechti, and Stefan Borgwardt**

**Document S1: Supplementary information**

**Content:**

Figure S1: Quality Checks with FSL's Squad
Figure S2: Group Differences in FA controlled for age and sex
Figure S3: Correlations between BDI and post-intervention FA values
Figure S4: Association between post-intervention scan delay and FA
Supplementary Tables
Table S1. Atlas Location and Number of Voxels Depicting Increased FA after LSD
Table S2. Univariate Test Results from the MANCOVA: Effects of Dosage, Pre-Intervention FA, Age, and Sex on Change in Depression and Mean FA Post-Intervention
Supplementary Resources
Data S1. Python Script for Individual Response Analysis and Visualization

**Figure S1:** Quality Checks with FSL's Squad

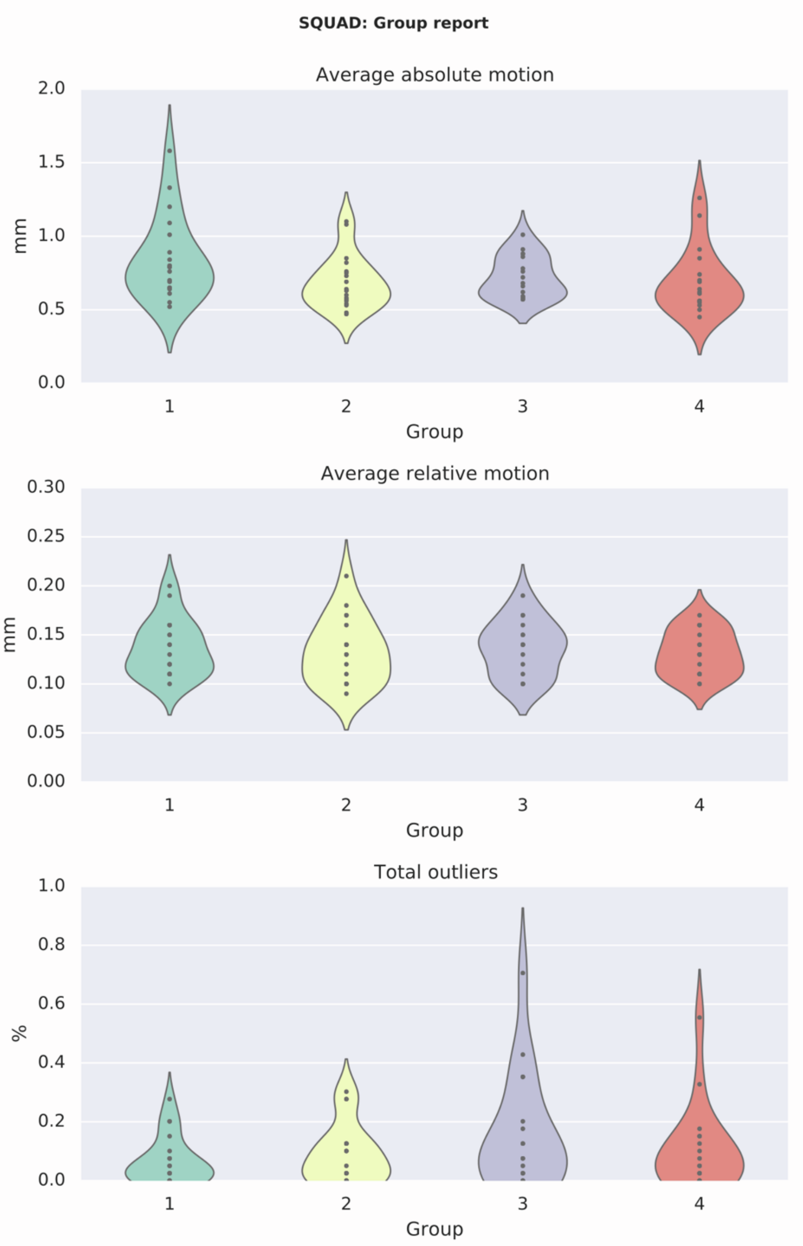


Depicted are the results of group quality check analysis (Squad). The groups (1 = LD-LSD post-intervention, 2 = LD-LSD pre-intervention, 3=HD-LSD post-intervention, 4= HD-LSD pre-intervention) did not differ significantly in average absolute and relative head motion or in the total number of outliers. Related to the section 'Preprocessing and Data Quality Check' in the STAR methods.

**Figure S2:** Group Differences in FA controlled for age and sex

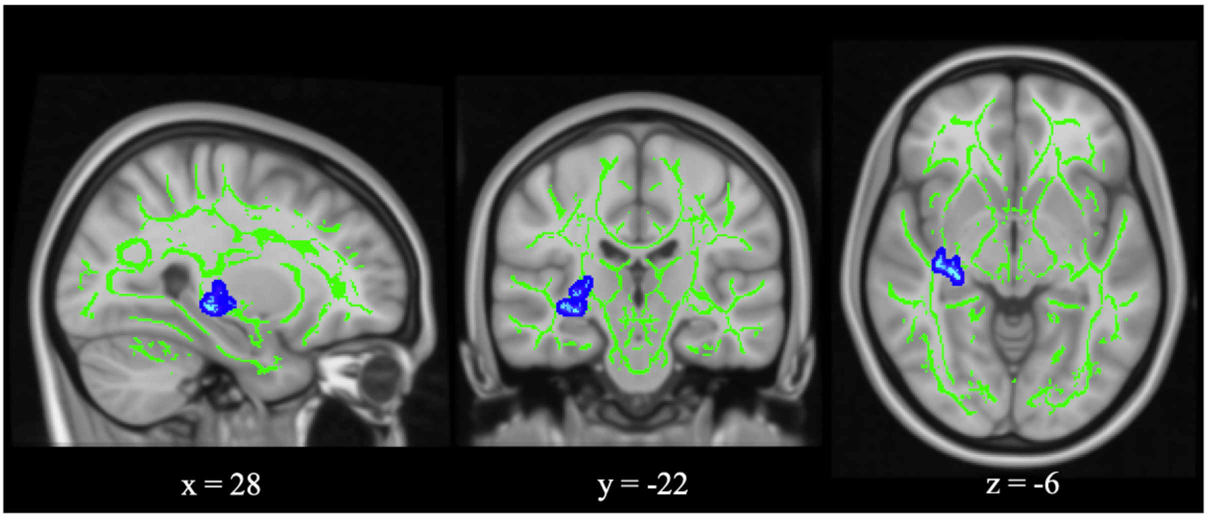


Depicted are the results of a control analysis (i.e., independent-sample t-test on the post-intervention-pre-intervention FA ‘difference images’ between the high-dose and low does LSD groups), in which we evaluated the influence of age and sex on our main result (i.e., increased FA in the HD-LSD group). Controlling for age and sex did not significantly change the results. The identified FA clusters at $P < 0.05$ depict increases in WM microstructure observed for the HD-LSD group in several regions (blue voxels). The mean FA skeleton (green) is overlaid on FSLeyes’s standard MNI152_T1_0.5mm template. MNI coordinates: x=28, y=-22, z=-6. *Abbreviations*: FA – fractional anisotropy, TFCE - threshold-free cluster enhancement, FEW- family-wise error, MNI - Montreal Neurological Institute. Related to the sections 'Control Analyses' in the STAR methods.

**Figure S3:** Correlations between BDI and post-intervention FA values

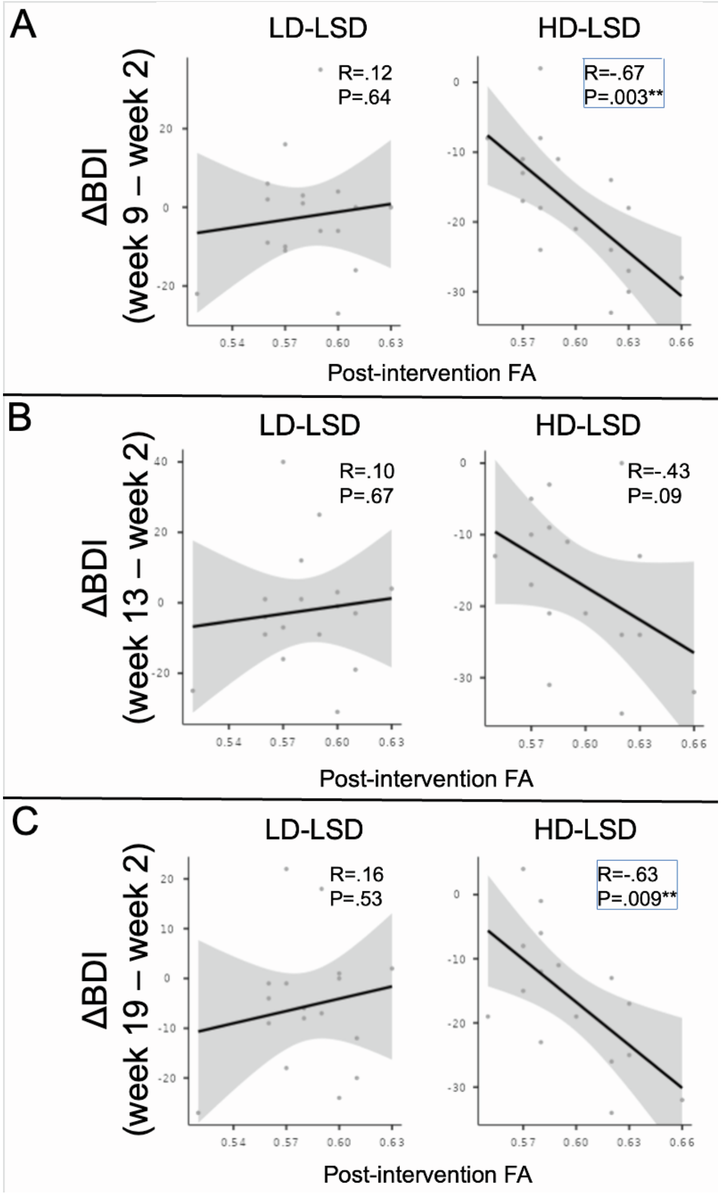


Depicted are correlations between changes from baseline (Δ) in the Beck Depression Inventory and post-intervention fractional anisotropy (FA) values in the areas identified by the independent-sample t-test for both the low (LD) and moderate-to-high dose LSD (HD-LSD) groups. (A) Depicted are correlations at the primary endpoint (week 9), (B) correlations at the first follow-up (week 13), and (C) correlations at the final follow-up (week 19). Blue frames around R and P values reflect results that remain significant after controlling for age and sex in partial correlation analyses. Related to Figure 3 and the Results section 'Correlations Between DTI-derived Measures and Clinical Scores'.

**Figure S4:** Association between post-intervention scan delay and FA

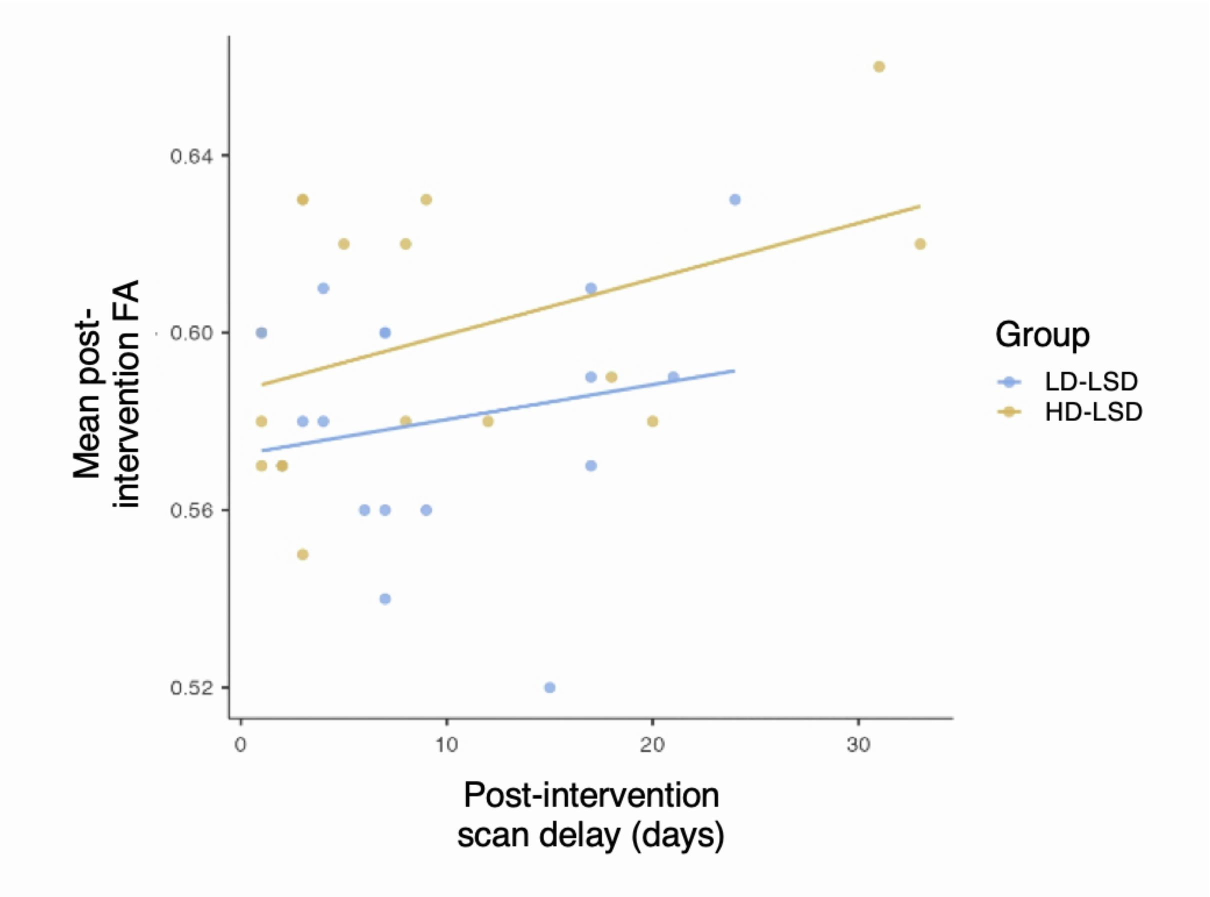


Scatterplot depicting the relationship between the number of days elapsed from the second intervention to the post-intervention MRI and FA values. An independent-samples t-test showed that the low-dose (LD-LSD) and moderate-to-high-dose (HD-LSD) groups did not differ in the duration between the second intervention and the second scan ($t_{33} = 0.01$; $p = 0.99$). (ii) A linear regression analysis using the number of days to scan as a predictor for post-intervention FA revealed a trend toward significance across groups ($F_{1,33} = 3.76$, $p = 0.06$), suggesting a possible relationship between FA increases and time elapsed since treatment. Individual data points and regression lines are displayed for both groups. Related to the 'Limitations of the Study' section in the main text.

**Supplementary Tables**

**Table S1.** Atlas Location and Number of Voxels Depicting Increased FA after LSD

| Region: JHU ROI | No. of voxels |
|---|---|
| Internal capsule: posterior limb | 3 |
| Internal capsule: retrolenticular | 38 |
| External capsule | 77 |
| Sagittal stratum | 39 |
| Fornix/ Stria terminalis | 65 |

Related to Figure 1 and the Results section 'Group-by-Time Interactions in DTI-derived Measures'.

**Table S2.** Univariate Test Results from the MANCOVA: Effects of Dosage, Pre-Intervention FA, Age, and Sex on Change in Depression and Mean FA Post-Intervention

| | Dependent Variable | Sum of Squares | df | F | P |
|---|---|---|---|---|---|
| **Dosage/ Group** | Change in depression | 2081.05882 | 1 | 12.087 | 0.002* |
| | Post-intervention FA | 0.00231 | 1 | 5.250 | 0.029* |
| **Pre-intervention FA** | Change in depression | 124.44697 | 1 | 0.723 | 0.402 |
| | Post-intervention FA | 0.00513 | 1 | 11.677 | 0.002* |
| **Age** | Change in depression | 388.59273 | 1 | 2.257 | 0.144 |
| | Post-intervention FA | 0.00656 | 1 | 14.927 | < .001* |
| **Sex** | Change in depression | 411.08911 | 1 | 2.388 | 0.133 |
| | Post-intervention FA | 6.60e-4 | 1 | 1.502 | 0.230 |
| **Residuals** | Change in depression | 4993.04765 | 29 | | |
| | Post-intervention FA | 0.01274 | 29 | | |

Table S2 presents the univariate test results derived from a MANCOVA examining the effects of dosage (HD-LSD/ LD-LSD), pre-intervention fractional anisotropy (FA), age, and sex on change in depression (i.e., ΔIDS-C at 2 weeks after the intervention) and mean FA post-intervention. Variables: The independent variables include dosage, pre-intervention FA, age, and sex. The dependent variables are change in depression and mean FA post-intervention. Univariate F-tests were conducted to assess the significance of each independent variable's effect on the dependent variables. Significant P-values are denoted by *. Related to the Results section 'Correlations Between DTI-derived Measures and Clinical Scores'.

**Table S3.** Associations between Subjective Experience, Clinical Scores, and FA

| | IDS-C (2 weeks) | IDS-C (6 weeks) | IDS-C (12 weeks) | Mean FA (post-intervention) |
|---|---|---|---|---|
| **Across Groups** | | | | |
| OB | R=-0.50, P=0.002** | R=-0.47, P=0.005** | R=-0.42, P=0.015* | R=0.05, P=0.76 |
| MEQ30 | R=-0.49, P=0.003** | R=-0.51, P=0.002** | R=-0.43, P=0.012* | R=0.15, P=0.36 |
| **HD-LSD** | | | | |
| OB | R=-0.38, P=0.13 | R=-0.52, P=0.038* | R=-0.49, P=0.054 | R=0.03, P=0.88 |
| MEQ30 | R=-0.38, P=0.12 | R=-0.59, P=0.016* | R=-0.51, P=0.042* | R=0.04, P=0.87 |
| **LD-LSD** | | | | |
| OB | R=-0.29, P=0.25 | R=-0.13, P=0.61 | R=-0.07, P=0.77 | R=-0.39, P=0.10 |
| MEQ30 | R=-0.17, P=0.50 | R=-0.05, P=0.84 | R=0.01, P=0.95 | R=-0.26, P=0.29 |

Pearson correlation coefficients and corresponding p-values are depicted between subjective effects, clinical scores, and post-intervention FA. *Abbreviations:* IDS-C: Inventory of Depressive Symptomatology Clinician-Rated; OB -oceanic boundlessness; MEQ30 – Mystical Experience Questionnaire total score; FA – fractional anisotropy. Related to the Results section 'Correlations Between DTI-derived Measures and Subjective Effects'.

**Table S4.** Partial correlations between post-intervention Mean FA and clinical improvement (IDS-C, IDS-SR, BDI), controlling for baseline depression severity (Week 2)

| **Time Point** | **$R_p$** | **P-value** |
|---|---|---|
| **IDS-C** | | |
| Primary (2w) | -0.41 | 0.11 |
| 1st Follow-up (6w) | -0.40 | 0.13 |
| 2nd Follow-up (12w) | -0.66 | 0.007* |
| **IDS-SR** | | |
| Primary (2w) | -0.44 | 0.087 |
| 1st Follow-up (6w) | -0.41 | 0.123 |
| 2nd Follow-up (12w) | -0.63 | 0.010* |
| **BDI** | | |
| Primary (2w) | -0.63 | 0.010* |
| 1st Follow-up (6w) | -0.29 | 0.28 |
| 2nd Follow-up (12w) | -0.56 | 0.028* |

The table depicts results for the High-Dose LSD group (n=17). Scores represent the change from baseline (week 2). * entries indicate P < 0.05. Related to the 'Limitations of the Study' section in the main text.

**Supplementary Resources**

**Data S1.** Python Script for Individual Response Analysis and Visualization

```
import pandas as pd

# Load participant FA data
# Expected CSV format: Patient ID, Group, Pre-FA, Post-FA
df = pd.read_csv("FA_file.csv")

# Reshape data to long format for easier processing
df_long = pd.melt(df,
                  id_vars=["Patient ID", "Group"],
                  value_vars=["Pre-FA", "Post-FA"],
                  var_name="Timepoint",
                  value_name="FA")

# Map group identifiers to descriptive labels
# Group 1 = Low dose/Placebo, Group 2 = Moderate-to-high dose (LSD)
df_long["Group"] = df_long["Group"].map({1: "Low dose", 2: "High dose"})

# Pivot back to wide format to calculate delta per individual
df_wide = df_long.pivot_table(index="Patient ID",
                              columns="Timepoint",
                              values="FA")

# Re-attach group information
group_info = df_long.drop_duplicates("Patient ID")[["Patient ID", "Group"]]
df_wide = df_wide.merge(group_info, on="Patient ID")

# Calculate Delta (Post-intervention minus Pre-intervention)
df_wide = df_wide.dropna(subset=["Pre-FA", "Post-FA"])
df_wide["delta"] = df_wide["Post-FA"] - df_wide["Pre-FA"]

# Print summary statistics for responders (Positive Delta)
for group in ["Low dose", "High dose"]:
    group_df = df_wide[df_wide["Group"] == group]
    total = len(group_df)
    positive = (group_df["delta"] > 0).sum()
    percent = 100 * positive / total
    print(f"{group}: {positive} of {total} ({percent:.1f}%) positive changes")
```

Related to Figure 2.
